# Properties of tin oxide films grown by atomic layer deposition from tin tetraiodide and ozone

**DOI:** 10.3762/bjnano.14.89

**Published:** 2023-11-13

**Authors:** Kristjan Kalam, Peeter Ritslaid, Tanel Käämbre, Aile Tamm, Kaupo Kukli

**Affiliations:** 1 Institute of Physics, University of Tartu, W. Ostwaldi 1, 50411 Tartu, Estonia

**Keywords:** atomic layer deposition, tin oxide, tin tetraiodide

## Abstract

Polycrystalline SnO_2_ thin films were grown by atomic layer deposition (ALD) on SiO_2_/Si(100) substrates from SnI_4_ and O_3_. Suitable evaporation temperatures for the SnI_4_ precursor as well as the relationship between growth per cycle and substrate temperature were determined. Crystal growth in the films in the temperature range of 225–600 °C was identified. Spectroscopic analyses revealed low amounts of residual iodine and implied the formation of single-phase oxide in the films grown at temperatures above 300 °C. Appropriateness of the mentioned precursor system to the preparation of SnO_2_ films was established.

## Introduction

Atomic layer-deposited SnO_2_ films have been studied from many perspectives. For example, one can mention anodes for Li-ion batteries [1], gas sensors [2], catalytic activities [3], and stable buffer [4] or base [5] layers in solar cells. More applications can be found, when SnO_2_ is considered as constituent of a nanostructure or a nanocomposite layer. ZrO_2_–SnO_2_ stacked layers have been shown to perform as mechanically elastic and magnetizable films [6]. SnO_2_-coated carbon nanotubes have been studied as catalysts [7] and ZnO–SnO_2_ as functional composite in Li-ion batteries [8]. A recent review article from 2022 lists 27 different precursor combinations for obtaining SnO_2_ in atomic layer deposition (ALD) processes [9]. Two of these processes have employed SnI_4_ as the metal precursor with either O_2_ [10–14] or H_2_O_2_ [10–11,15] as oxidizer.

Of these two oxygen sources, O_2_ would be more desirable because with it a hydrogen-free process is possible, which means less contamination and residuals in the films. Another advantage of the O_2_ process is a maximum growth per cycle (GPC) three times higher than in the H_2_O_2_ process [10]. The drawback of the O_2_ process is a relatively high deposition temperature, starting from 400 °C [10–14] and achieving the maximum GPC of about 0.12 nm/cycle around 600 °C [13–14]. To date, a process using SnI_4_ and O_3_ has not been published. The present paper shows that using O_3_ instead of O_2_, the deposition temperature can be brought down to 225 °C and the GPC can be enhanced. The authors aim to provide a comprehensive description of said process and the resulting films.

## Experimental

The ﬁlms studied in this work were grown in a low-pressure ﬂow-type ALD reactor [16]. Tin(IV) iodide, SnI_4_ (99.999%, Sigma-Aldrich), used as the tin precursor was evaporated at 83 °C from a half-open glass boat inside the reactor. Nitrogen, N_2_ (99.999%, AS Linde Gas), was applied as the carrier and purging gas. Ozone, produced from O_2_ (99.999%, AS Linde Gas), was used as oxidizer, with a concentration of 220–250 g/m^3^. The ALD process was carried out in the temperature range of 100–600 °C when investigating the dependence of different film properties on the deposition temperature. Other experiments were carried out at 300 °C since the highest GPC, which was 0.27 nm/cycle, was obtained at this temperature. In order to visualize the stepwise film growth and, at the same time, determine the optimum pulse length for the iodide precursor, the SnI_4_–O_3_ process was, at first, examined in situ using a quartz crystal microbalance (QCM) [17]. The QCM data were acquired with a Q-pod quartz crystal monitor (Inficon) at a stabilized reactor temperature of 300 °C. For the film growth for ex situ measurements, the cycle times for SnO_2_ were kept at 5-2-5-5 s, respectively, for the following sequence: metal precursor pulse, N_2_ purge pulse, O_3_ pulse, and N_2_ purge pulse. The films were grown on Si(100) cleansed and etched prior to the growth.

An X-ray fluorescence spectrometer Rigaku ZSX 400 with the program ZSX Version 5.55 was used to measure the elemental composition of films. A spectroscopic ellipsometer, model GES5-E, was used for the measurements of film thicknesses and refractive indices. Ellipsometric data was modelled using the Cauchy dispersion model. The crystal structure was evaluated by grazing incidence X-ray diffractometry (GIXRD), using an X-ray diffractometer SmartLab Rigaku with Cu Kα radiation, which corresponds to an X-ray wavelength of 0.15406 nm.

X-ray photoelectron emission and X-ray absorption spectroscopy (XPS and XAS, respectively) measurements were made at the FinEstBeAMS beamline [18] at a solids research endstation [19]. XPS was carried out using a SPECS Phoibos150 hemispherical photoelectron kinetic energy analyser at an overall spectral resolution of 0.3 eV. XAS was carried out at 0.1 eV spectral resolution in total electron yield (TEY) mode by measuring sample photocurrent and normalising the signal to a reference photocurrent signal from a clean gold mesh located behind the last optical element of the beamline.

## Results and Discussion

**Figure 1:**
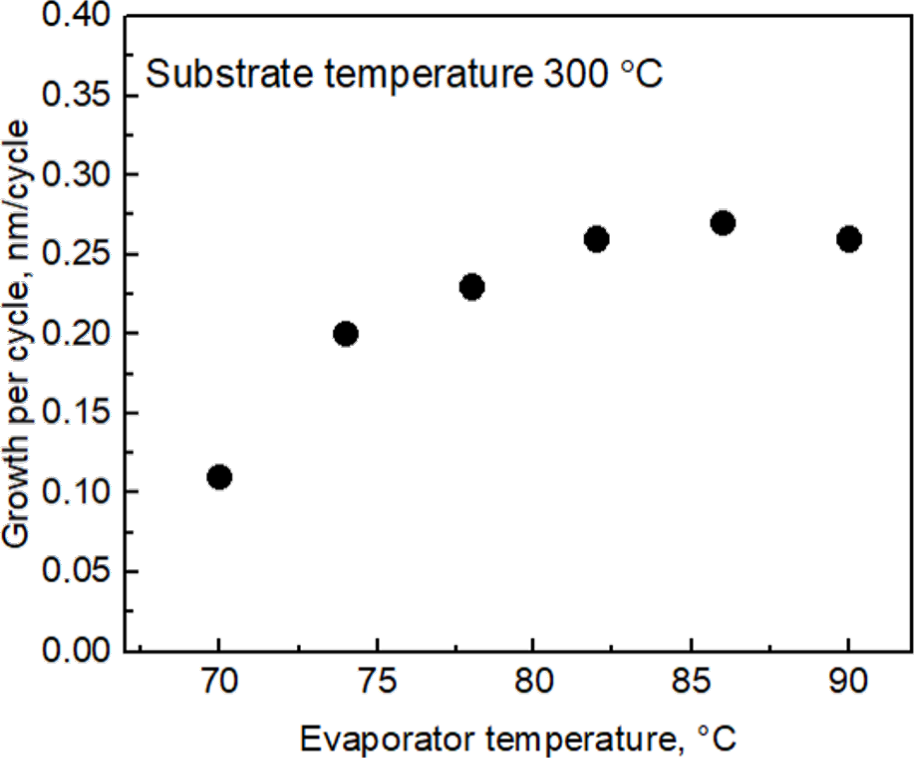
Dependence of SnO_2_ film GPC on the evaporation temperature of the SnI_4_ precursor. The cycle times were set at 5-2-5-5 s for the sequence SnI_4_ pulse, purge, O_3_ pulse, and purge.

To establish the evaporator temperature that provides the maximum coverage of substrate surface with precursor molecules and, correspondingly, the maximum growth rate, the dependence of GPC on the SnI_4_ evaporation temperature was examined. One can see in Figure 1 that the film GPC considerably increased with the evaporation temperature up to approximately 82 °C. Hence, the SnI_4_ evaporation temperature was set at 83 °C for further experiments.

**Figure 2:**
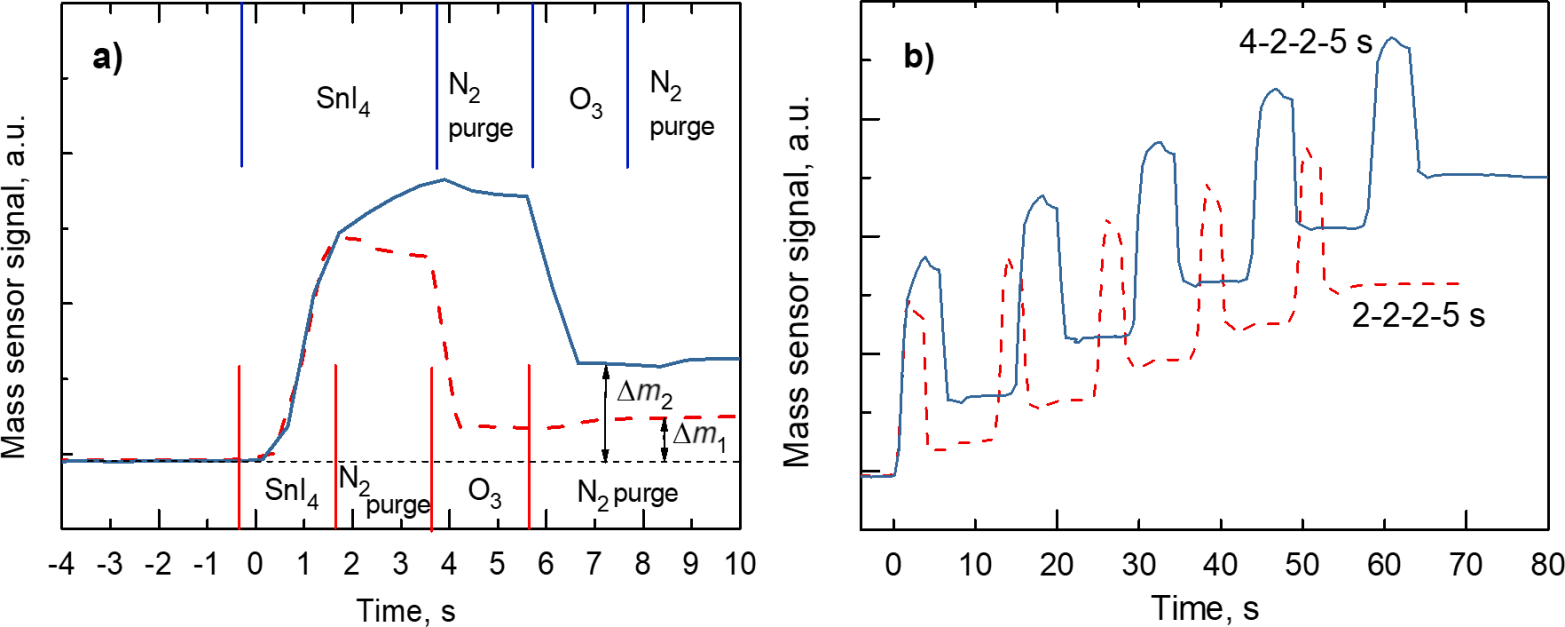
Time evolution of the QCM mass sensor signal during five sequential ALD cycles (a), and during one single ALD cycle (b). The labels “4-2-2-5 s” and “2-2-2-5 s” denote the pulse lengths in the sequence SnI_4_, N_2_, O_3_, and N_2_. ∆*m*_1_ and ∆*m*_2_ are the mass increments after a single ALD cycle with pulse lengths of 2-2-2-5 and 4-2-2-5 s, respectively. The mass sensor signal in arbitrary units is directly correlated to the increment in the QCM oscillation period.

Concurrently with the determination of the film thickness via ex situ measurements, the film growth upon cycling precursor pulses and purge periods was monitored in real time. The in situ monitoring helped in the visualization of the stepwise growth process (Figure 2). One can see that the application of sequential ALD cycles resulted in a continuous growth of solid film material, expressed by the mass sensor signal in arbitrary units.

**Figure 3:**
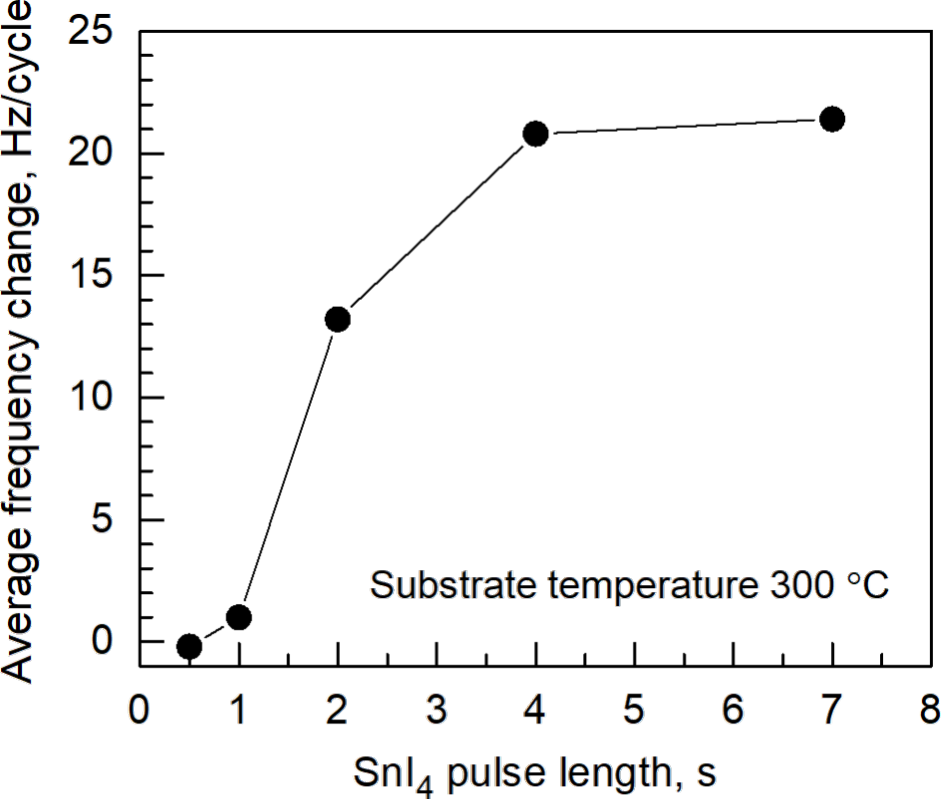
QCM frequency decrement measured per single ALD cycle as function of the SnI_4_ pulse length. The lengths of purge, O_3_, and purge pulses following the SiI_4_ pulse were 2, 5, and 5 s, respectively.

Monitoring the film growth by means of the QCM allowed for a fast determination of the metal precursor pulse length required for a nearly self-saturating adsorption process and maximized growth during a single cycle. It has, however, to be noted that a clear self-saturating adsorption process of the metal precursor was not recognized because the QCM signal did not fully stabilize at any metal precursor exposure time but continued to increase (Figure 2). A drop in the QCM signal during O_3_ pulses accompanies the release of relatively heavy iodine, and the QCM signal stabilised after the completion of the oxidation step (Figure 2). Figure 3 depicts the change in the oscillation frequency as function of the SnI_4_ pulse length. The latter tests revealed that a SnI_4_ exposure time of 5 s is sufficient for effective coverage of the surface with adsorbed species. In the same manner, through varying the O_3_ pulse length an optimal pulse length was derived (not shown here).

**Figure 4:**
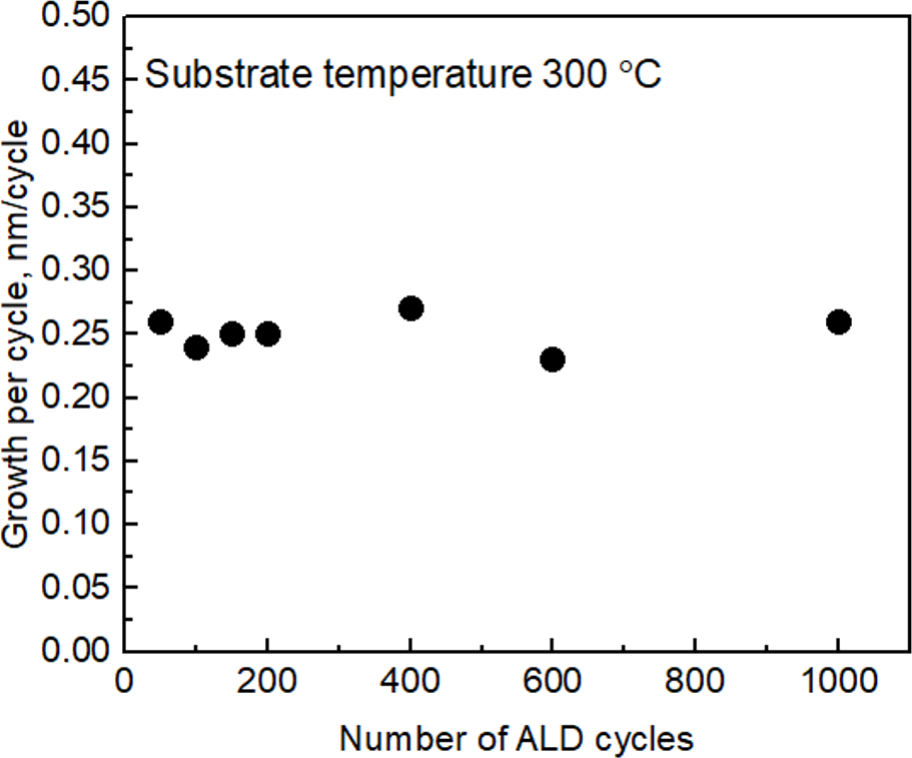
SnO_2_ film GPC as function of the number of ALD cycles.

Varying the number of ALD cycles showed that there was no significant incubation period at the early stage of the ALD process (Figure 4).

**Figure 5:**
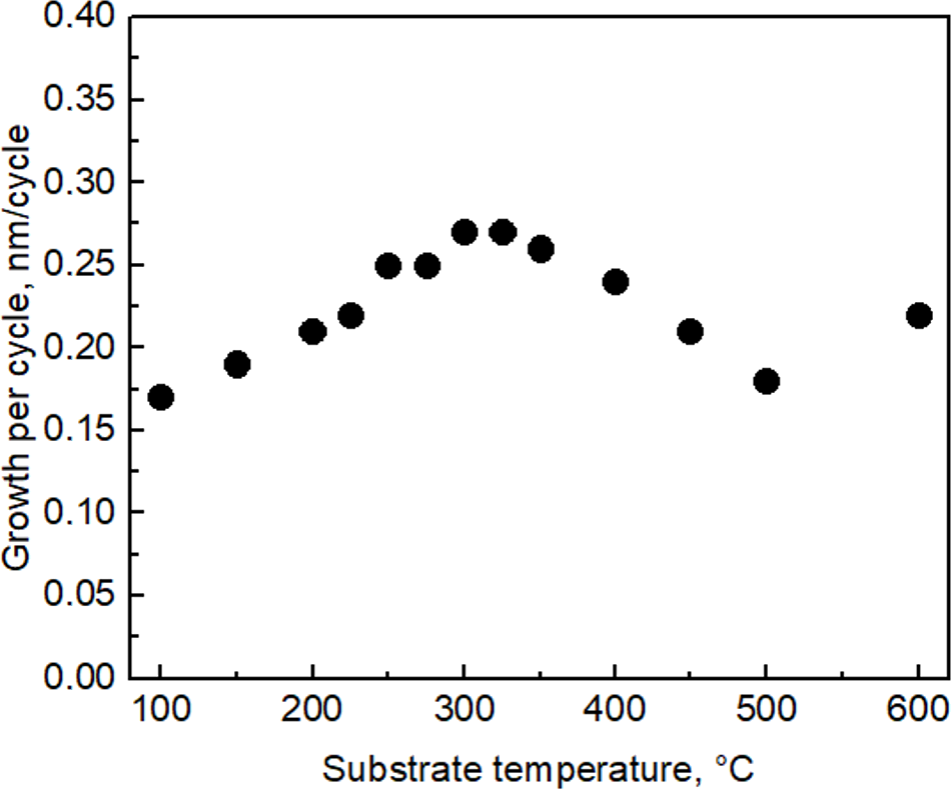
SnO_2_ GPC as function of the substrate temperature.

The highest GPC of the films was obtained at a substrate temperature of 300 °C (Figure 4). Obviously, there was no significant temperature window for saturation [20], that is, the so-called ALD window, in any temperature range. This may be related to the partial decomposition of precursor molecules during the adsorption step of the first precursor and the concurrently intensified release of ligand, that is, iodine molecules, I_2_. Earlier, analogous studies have been carried out on TiO_2_ films grown by ALD using TiI_4_ and O_2_ as precursors [21]. Plausibly, the initial increase in the GPC up to 300 °C is caused by the gradually enhancing decomposition of metal iodide on the receiving surface, which is not to be regarded as self-saturating adsorption process. Nevertheless, in the adsorption step, more metal is added to the growing layer upon increasing the substrate temperature. Hence, during the purge period after the metal precursor pulse, one could record a decrement in the mass adsorbed on the surface in the present study (Figure 2), as well as in the earlier studies on TiI_4_-based ALD of TiO_2_ [21]. The decrement of the mass adsorbed during the precursor pulse can be explained by the desorption of iodine from the surface, the desorption rate of which increases upon increasing temperature. It is thus reasonable to believe that the decreasing GPC of the SnO_2_ films above 300 °C (Figure 5) is caused by the increasing rate of I_2_ desorption before the solid metal oxide could be formed by the reaction of surface iodide species with ozone. Further, the apparent increase in the GPC at the highest temperature examined (600 °C) is probably caused by an uncontrolled decomposition of the precursor, also related to the enhancement of the lateral film thickness profile along the gas flow direction.

**Figure 6:**
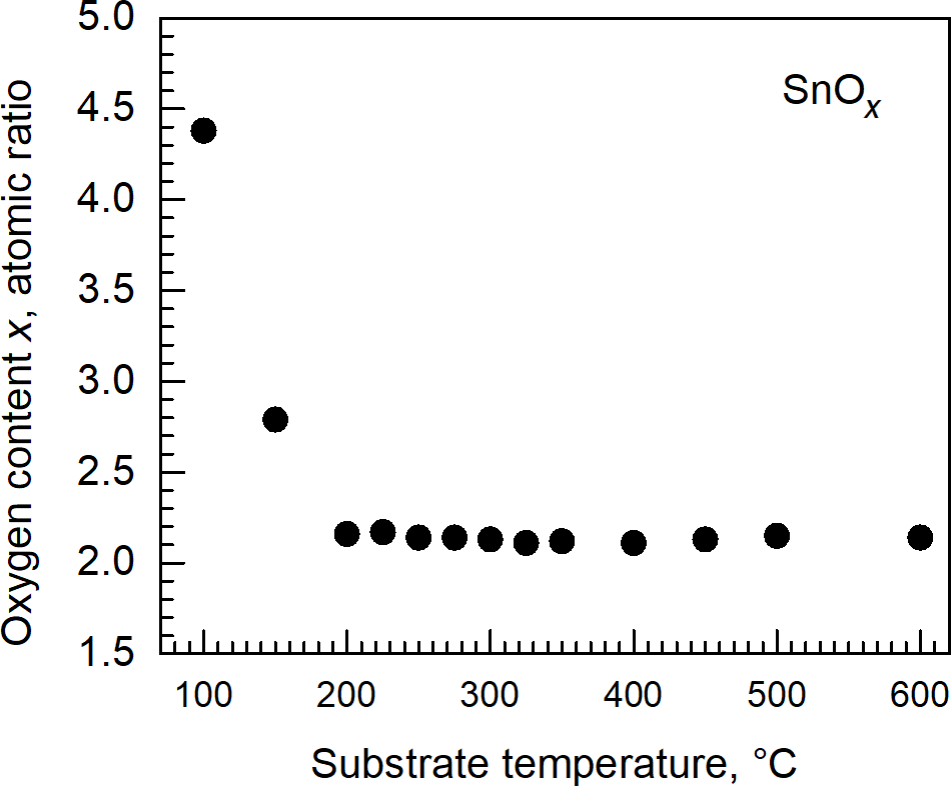
Oxygen content in the SnO_2_ films as function of the substrate temperature.

**Figure 7:**
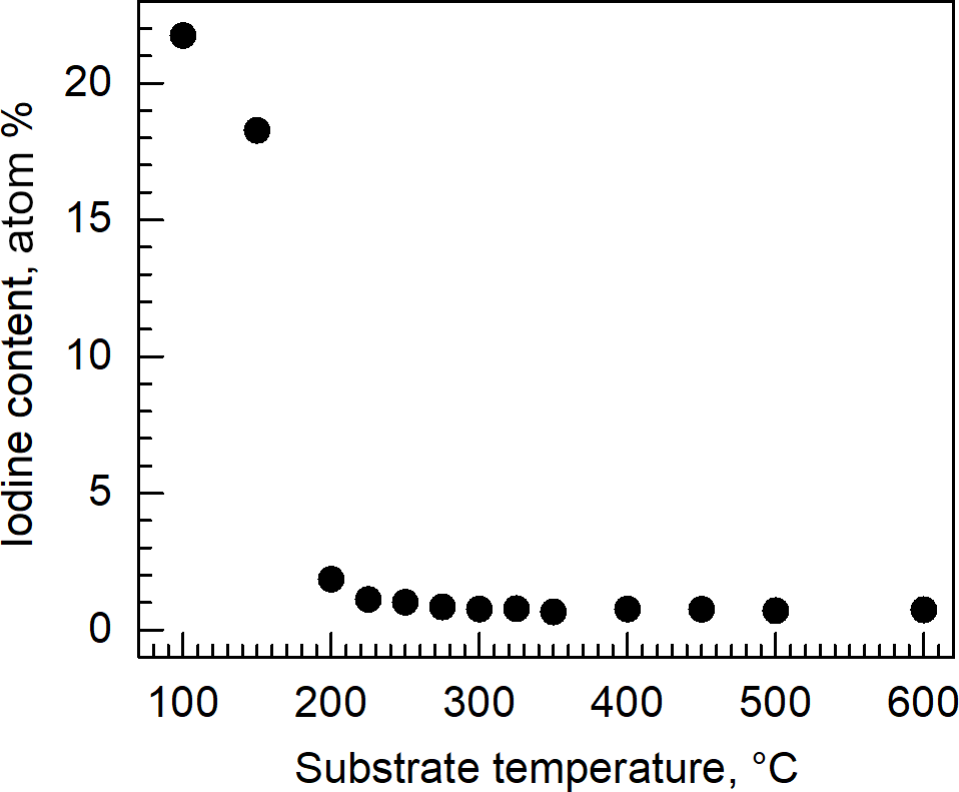
Content of residual iodine in tin oxide films deposited from tin iodide and ozone in the temperature range of 100–600 °C.

Analysis of oxygen and iodine contents in the films revealed that above a substrate temperature of 200 °C, the oxygen content remained stable. At temperatures below 200 °C, the oxygen content was significantly higher than that expected from a stoichiometric metal dioxide (Figure 6). A similar behaviour was observed for the iodine content. The films grown at temperatures below 200 °C were characterized by a markedly high iodine content (Figure 7). The iodine content decreased upon increasing the deposition temperature above 200 °C and, above 300 °C, stabilized at an appreciably low level of 0.7–0.8 atom % (Figure 7). Since the relatively high oxygen content below a deposition temperature of 200 °C did not arise from increased oxygen amounts in the film, but from decreased tin amounts, one can propose that, at the lowest deposition temperatures, I_2_O_5_ forms as a significant component in addition to the SnO_2_ host. Hence, a functional SnO_2_ film is most likely not realizable in this process below 200 °C. Even if formed, I_2_O_5_ would decompose at temperatures above 300 °C [22]. In our study, iodine and oxygen levels drop to the values low enough to obtain stoichiometric SnO_2_ as the major phase at temperatures higher than 300 °C.

### Film structure

**Figure 8:**
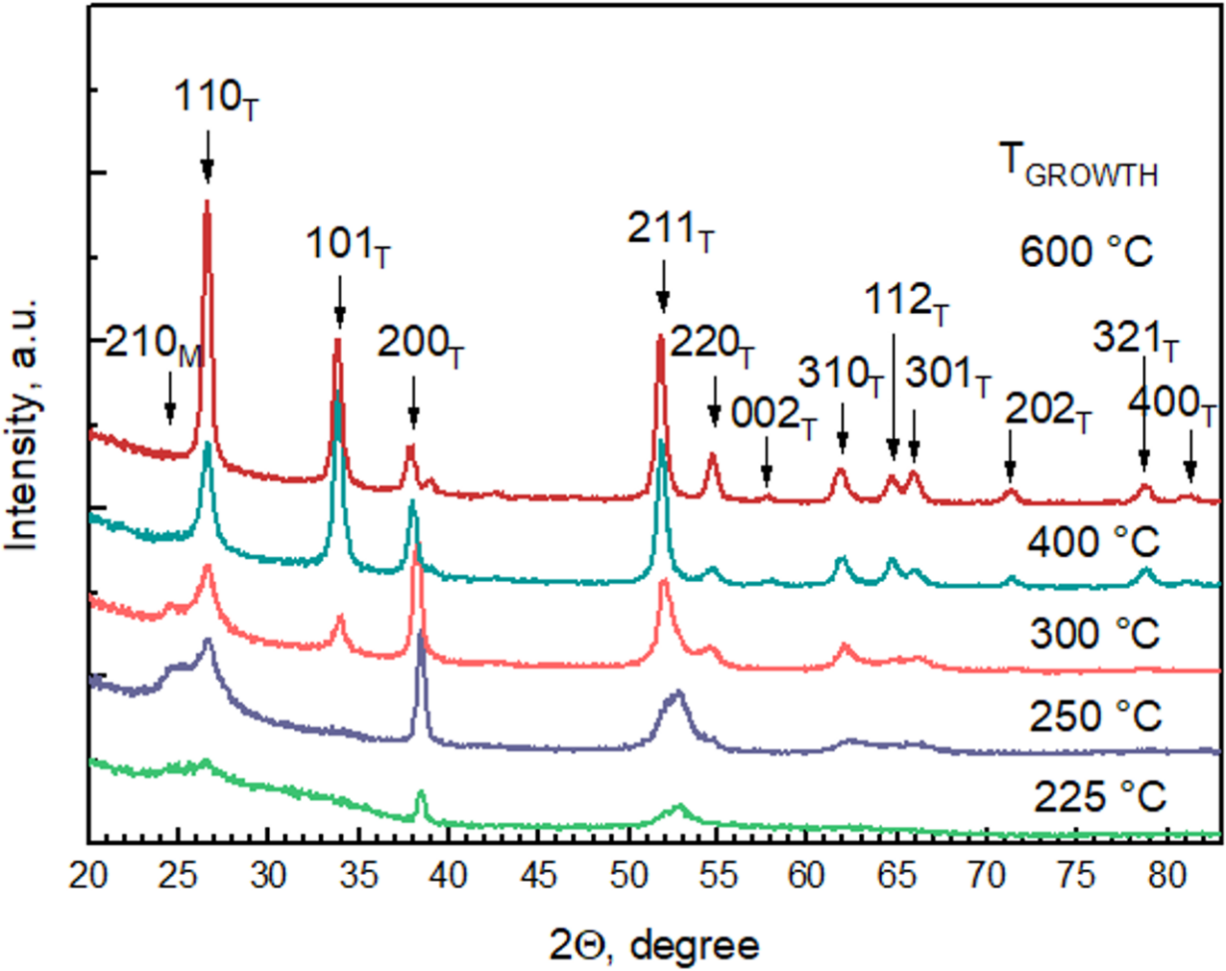
GIXRD patterns of SnO_2_ films deposited at the given temperatures. Miller indices attributed to the diffraction maxima are indicated on the graph, where T corresponds to the tetragonal crystalline phase of SnO_2_ and M corresponds to the monoclinic phase of I_2_O_5_.

Increasing the deposition temperature increased the crystallinity of films, as can be seen in Figure 8. Diffractograms of all crystalline films revealed the presence of tetragonal SnO_2_ (PDF Card 01-071-5324). Depositions were carried out starting from 100 °C; however, XRD patterns from the films deposited at the lowest temperatures of 100–200 °C are not depicted in Figure 8 since the films grown at 225 °C were the first that revealed distinguishable reflections. The latter likely means that a significant portion of the films deposited below 200 °C is not SnO_2_. One diffraction maximum at 24.5° that exists in samples up to 300 °C is not attributable to any SnO_2_ phase. This is, however, the diffraction maximum of monoclinic I_2_O_5_ (PDF card 00-022-0338). This is consistent with the higher iodine and oxygen content measured in films deposited at lower temperatures.

**Figure 9:**
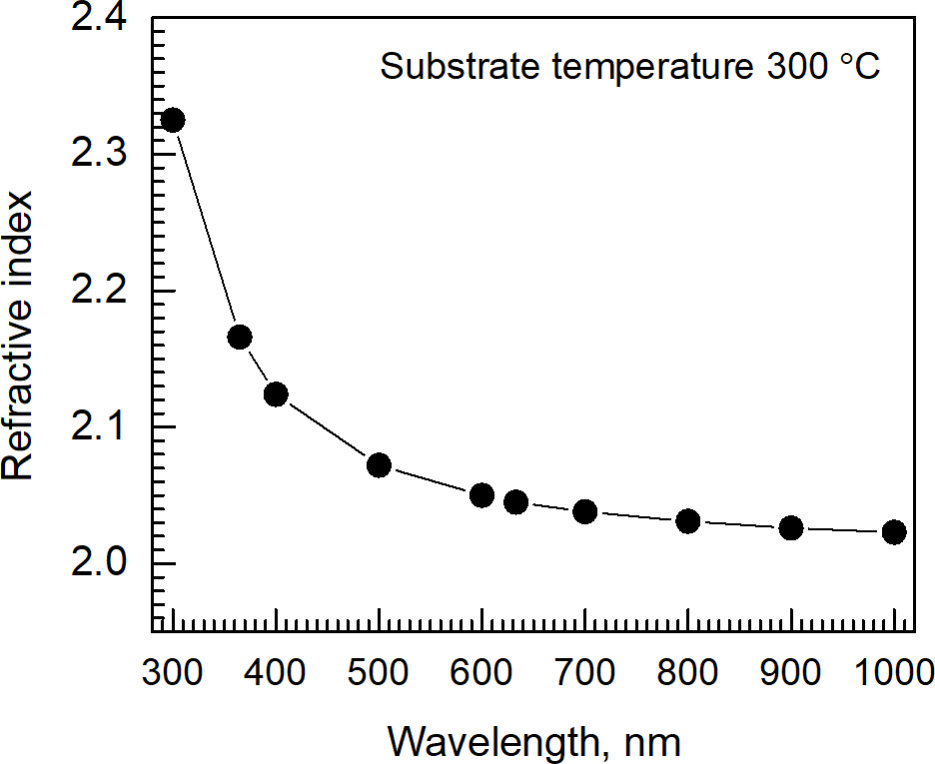
Refractive index of a SnO_2_ film deposited at 300 °C to a thickness of 110 nm as function of the wavelength.

The refractive indices of the films were stable throughout the sample series with different deposition temperatures, remaining between 2.04 and 2.06 at 633 nm wavelength for predominantly SnO_2_ films with little residuals. One representative dispersion curve from a film deposited at 300 °C is presented in Figure 9.

The refractive index values in the range of 2.04–2.06 can be regarded as appreciably high for SnO_2_ thin films. Refractive index values of approx. 2.0 were measured by ellipsometry for SnO_2_ films grown by ALD from bis(1-dimethylamino-2-methyl-2-propoxide)Sn as the Sn precursor and either H_2_O plasma or O_2_ plasma as the oxygen source [23]. Refractive index values between 2.0 and 2.1, comparable to those measured in the present study, have been obtained in a paper reporting the results of thermal ALD of SnO_2_ from tetrakis(dimethylamino)tin as the Sn precursor and ozone as the oxygen source [24]. The refractive index values obtained in the present study exceed those measured from SnO_2_ films deposited using spray pyrolysis [25] but remain inferior to those of post-growth-annealed SnO films grown via successive ionic layer adsorption and reaction [26].

**Figure 10:**
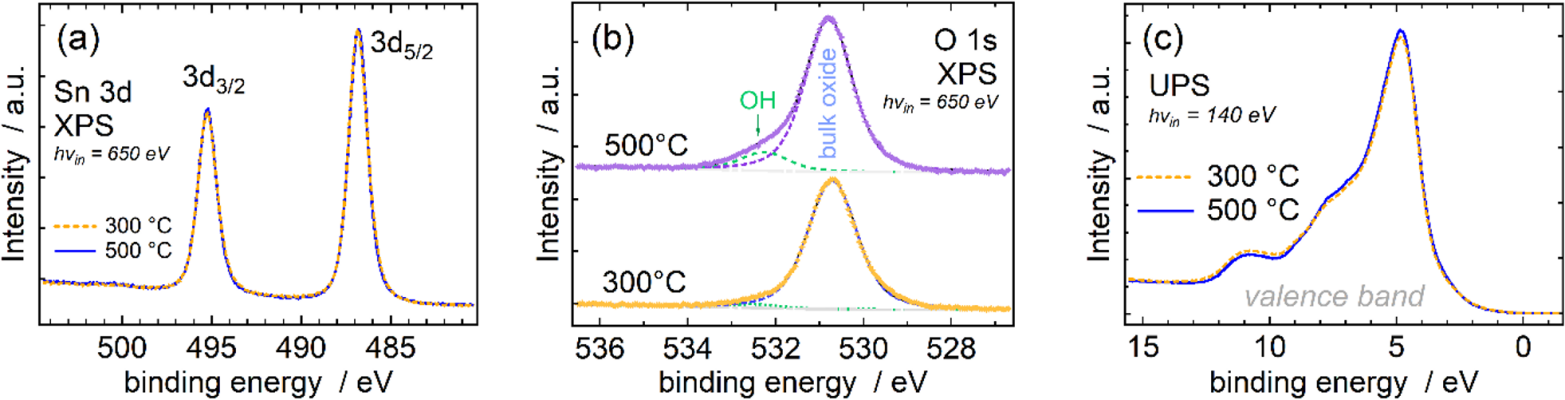
XPS results of (a) Sn 3d, (b) O 1s, and (c) valence-band region of the SnO_2_ films deposited at 300 and 500 °C.

The SnO_2_ films grown at 300 and 500 °C, that is, those with low residual iodine content, were analysed ex situ in terms of surface chemistry using soft X-ray spectroscopy methods. The Sn 3d XPS data (Figure 10a) show almost identical spectra for the samples deposited at 300 and 500 °C, and the narrow Sn 3d line profiles suggest the formation of a single-phase compound. The 2+ and 4+ charge states possible for Sn in its oxides have only a small (approx. 0.8 eV) difference in binding energy, which is less than the linewidth (FWHM ≈ 1.2 eV) of either compound [27–28]. Therefore, for mixed-phase compounds an asymmetric widening of the overall line profiles is observed rather than the appearance of additional resolved peaks [27–28]. The Sn 3d_5/2_ XPS binding energy of 486.9 eV in our data also aligns well with stoichiometric SnO_2_ as reported previously [27–34], indicating a single-phase Sn 4+ compound in our samples.

In O 1s XPS (Figure 10b), the dominant peak at 530.7 eV also aligns well with values earlier reported for SnO_2_ [32–34]. A bit surprisingly, the spectra obtained from the sample grown at the higher deposition temperature revealed a somewhat more intense peak at the binding energy typical of surface OH (Figure 10b). This might be related to the chemisorption and decomposition of environmental humidity, enhanced on more crystallised film surfaces. Such a surface OH contribution has been described earlier [33–34].

Complementarily, the valence-band photoelectron spectra (Figure 10c) closely resemble those reported for SnO_2_ [28] with a dominant peak just below 5 eV and further distinct features at around 7.5 and 11 eV. This is in rather stark contrast to the SnO valence band, where mainly the occupied Sn 5s states give rise to a significant peak at ca. 2.5 eV binding energy, whereas the peak at 5 eV is not as dominant [28–29,35].

**Figure 11:**
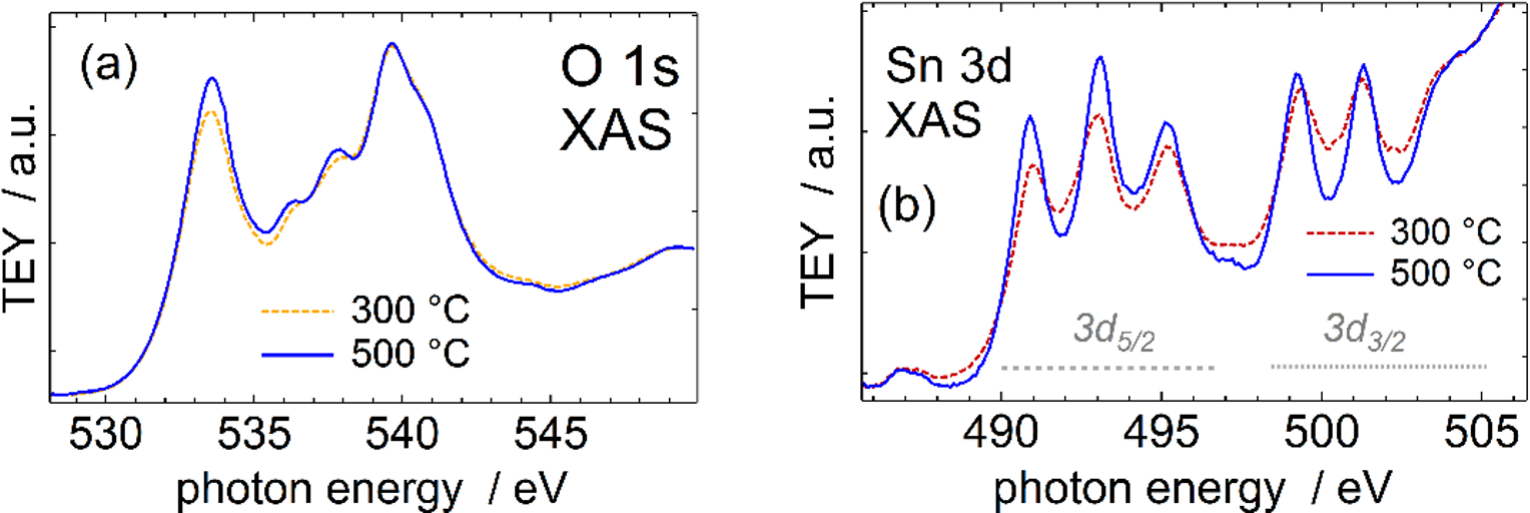
XAS results depicting O 1s (a) and Sn 3d (b) spectra of the SnO_2_ films deposited at 300 and 500 °C.

X-ray absorption spectra (Figure 11) were additionally recorded to possibly detect any differences between the samples. The Sn 3d XAS band is constituted by transitions from the 3d core orbital to the unoccupied p and f symmetry states, as defined by the dipole selection rules common for optical (first-order) transitions (Δ*l* = ±1), whereas the O 1s XAS probes the oxygen p character states [36].

The lowest excitation energy peak (at 533.6 eV) in the SnO_2_ O 1s XAS (Figure 11a) has been assigned to unoccupied O 2p states hybridised with ligand Sn 5s states, while the three consecutive peaks at approximately 536.3, 537.7, and 539.6 eV correspond to states hybridised with Sn 5p states [37–38]. In the SnO_2_ rutile structure, the latter states are non-degenerate because of non-equidistant ligands for the axes of the coordination octahedron and the linking direction of these octahedra. We note that any eventual contribution from SnO (i.e., due to non-complete oxidation at lower deposition temperatures [39–40]) would show a quite different O 1s absorption spectrum with the lowest energy maximum downshifted by ca. 1 eV [28–29,35–36,41] and a broader and less intense feature in the region corresponding to the Sn 5p states, which then corresponds to an e_g_ state split in *C*_4_*_v_* symmetry [37].

In the Sn 3d XAS results (Figure 11, right panel), the Sn 5s states are dipole-forbidden for transitions starting from the Sn 3d core level, whereas the triply split b_1u_, b_2u_ and, b_2u_ unoccupied states of Sn 5p origin in the *D*_2_*_h_* ligand symmetry, resulting in the three peaks at approximately 491, 493, and 495 eV (Figure 11b) in the Sn3d_5/2_ region, are characteristic of SnO_2_ [28–29,37,39,41]. The SnO Sn 3d spectrum is quite different with the Sn 3d_5/2_ main peak at almost 4 eV lower excitation energy (ca. 487 eV) [29,40–41] and no distinct triple peak structure, while the highest of the SnO_2_ triplet would already overlap with the SnO spin–orbit component (the Sn 3d_3/2_) main peak. In our opinion, the somewhat less sharp spectral shape and the slightly elevated relative intensity ratio of the 495 eV peak compared to the lower-energy 3d_5/2_ (491 and 493 eV) peaks suggests a minor SnO component present in the sample deposited at 300 °C. Alternatively, it is just a less structurally homogeneous sample, where slightly varying ligand distances average to less sharp XAS peaks.

Because of the somewhat less shallow probe depth of XAS recorded in TEY mode (ca. 10 nm) compared to the high surface sensitivity of the recorded photoemission spectra (a few atomic layers) [41] we suggest that less completely oxidised species appear below the outmost surface of the film.

## Conclusion

SnO_2_ thin films were deposited from SnI_4_ and O_3_ via ALD in the temperature range of 100–600 °C. The resulting films formed in the crystalline tetragonal phase of SnO_2_ when the deposition temperature was over 225 °C, and the proportion of crystallised material in the film grew with the deposition temperature. Films grown at 200 °C and lower temperatures did not exhibit ordered structure and contained high amounts of residual iodine. Also, the oxygen fraction was larger than that expected from stoichiometric SnO_2_. Starting from about 300 °C, the precursor chemistry applied and reported in this work provides a fast route to SnO_2_ films with clearly defined structural properties. The growth per cycle of the films was about 0.25 nm/cycle. The deposition temperature can be lower than the minimally 400 °C needed for a process using SnI_4_ and O_2_, and the growth per cycle is about two times larger than for a process using SnI_4_ and O_2_.

## Supporting Information

File 1 Supplementary material.
