# Supplementary material for: Properties of tin oxide films grown by atomic layer deposition from tin tetraiodide and ozone
Source: Beilstein J Nanotechnol. 2023 Nov 13;14:1085–92. doi: 10.3762/bjnano.14.89 (PMC10667712; doi:10.3762/bjnano.14.89)
Supplement: File 1 — Supplementary material. [file Beilstein_J_Nanotechnol-14-1085-s001.pdf]

BEILSTEIN JOURNAL OF NANOTECHNOLOGY

Supporting Information

for

Properties of tin oxide films grown by atomic layer deposition from tin tetraiodide and ozone

Kristjan Kalam, Peeter Ritslaid, Tanel Käämbre, Aile Tamm and Kaupo Kukli

Beilstein J. Nanotechnol. **2023,** *14,* 1085–1092. doi:10.3762/bjnano.14.89

Supplementary material

License and Terms: This is a supporting information file under the terms of the Creative Commons Attribution License (https://creativecommons.org/licenses/by/4.0). Please note that the reuse, redistribution and reproduction in particular requires that the author(s) and source are credited and that individual graphics may be subject to special legal provisions.
The license is subject to the *Beilstein Journal of Nanotechnology* terms and conditions: (https://www.beilstein-journals.org/bjnano/terms)

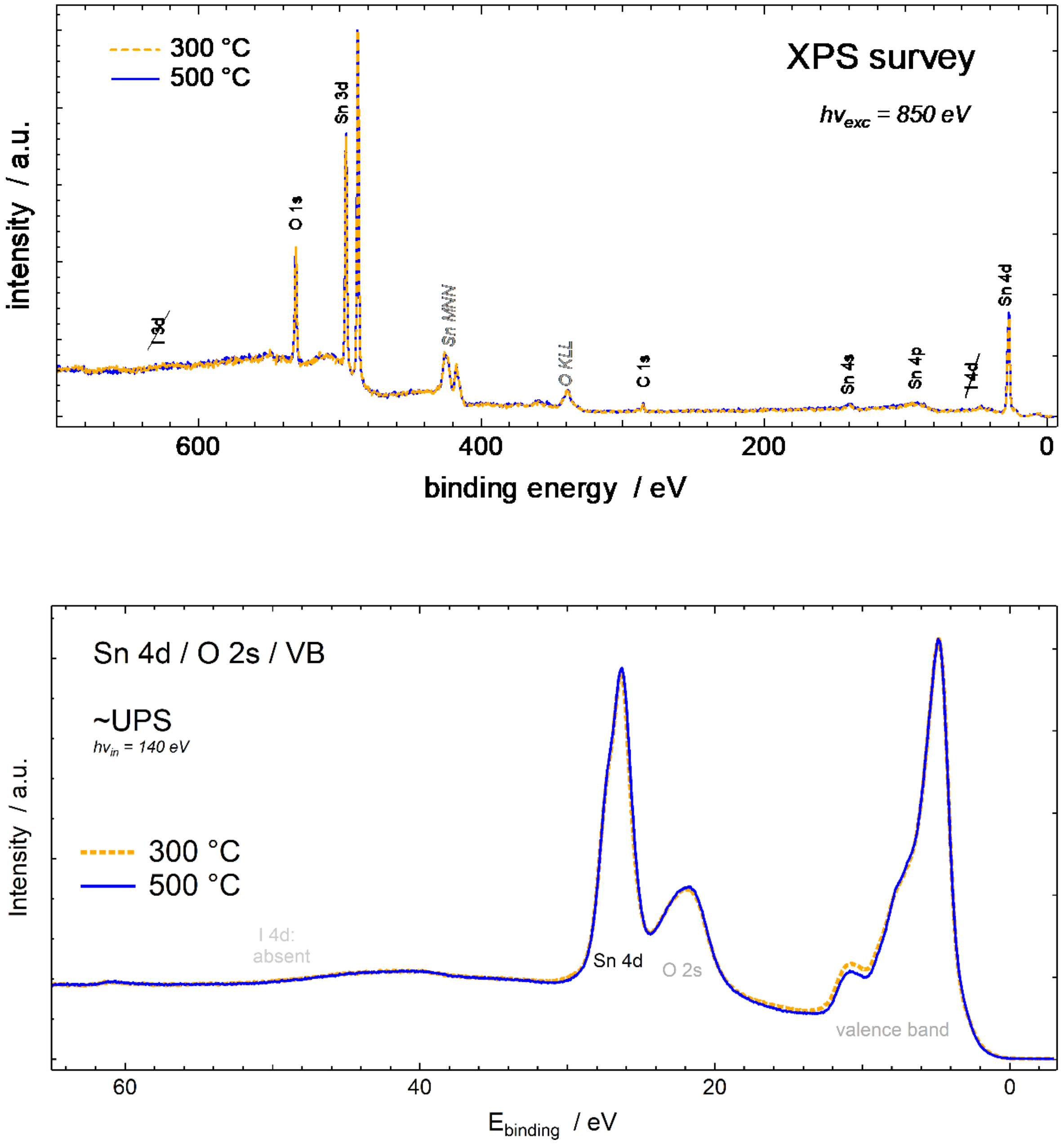

Figure S1: Top panel: XPS survey spectra visualising the virtually coinciding tin/oxygen content ratio and the absence of any residual iodine compounds even in the 300 °C sample. Bottom panel: an extended valence-band (and shallow core) binding energy region photoelectron spectrum. We note that the kinetic energy of the I 4d electrons is different in these two panels (ca. 800 vs. ca. 90 eV); therefore, also the probe depth differs. The inelastic mean free path of the photoelectrons changes from ca. 1.5 to 0.5 nm.

This can be compared to the truly bulk-sensitive XRD results, which indicate some iodine present in the 300 °C sample. A plausible explanation would be that although iodine sublimates from the surface region even at 300 °C (and is therefore not seen in XPS), it is not as mobile to segregate to the surface at this temperature (and appears therefore as present in the XRD data).

The C 1s XPS in Figure S2 is taken as a reference to assure that the sample is not charging. It indicates only a very limited adventitious carbon content and, for example, no alien carbonate species.

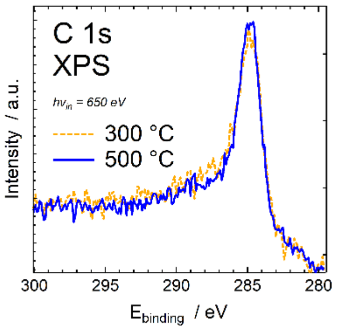

Figure S2: C 1s XPS results.
